# Nilvadipine suppresses inflammation via inhibition of P-SYK and restores spatial memory deficits in a mouse model of repetitive mild TBI

**DOI:** 10.1186/s40478-020-01045-x

**Published:** 2020-10-19

**Authors:** Alexander Morin, Benoit Mouzon, Scott Ferguson, Daniel Paris, Mackenzie Browning, William Stewart, Mike Mullan, Fiona Crawford

**Affiliations:** 1 The Roskamp Institute, Sarasota, FL USA; 2 The Open University, Milton-Keynes, UK; 3 James A Haley Veterans Administration, Tampa, FL USA; 4 Queen Elizabeth University Hospital, Glasgow, UK

## Abstract

Repeated exposure to mild TBI (mTBI) has been linked to an increased risk of Alzheimer’s disease (AD), chronic traumatic encephalopathy (CTE) and other neurodegenerative diseases. Some pathological features typically observed in AD have been found in postmortem brains of TBI and CTE, hence treatments tested for AD have a potential to be effective against r-mTBI outcomes. Neuroinflammation may present a possible answer due to its central role both in acute brain injury and in chronic degenerative-like disorders. Our previous studies have shown that drug nilvadipine, acting as an inhibitor of spleen tyrosine kinase (SYK), is effective at reducing inflammation, tau hyperphosphorylation and amyloid production in AD mouse models. To demonstrate the effect of nilvadipine in the absence of age-related variables, we introduced the same treatment to young r-mTBI mice. We further investigate therapeutic mechanisms of nilvadipine using its racemic properties. Both enantiomers, (+)-nilvadipine and (−)-nilvadipine, can lower SYK activity, whereas (+)-nilvadipine is also a potent L-type calcium channel blocker (CCB) and shown to be anti-hypertensive. All r-mTBI mice exhibited increased neuroinflammation and impaired cognitive performance and motor functions. Treatment with racemic nilvadipine mitigated the TBI-induced inflammatory response and significantly improved spatial memory, whereas (−)-enantiomer decreased microgliosis and improved spatial memory but failed to reduce the astroglial response to as much as the racemate. These results suggest the therapeutic potential of SYK inhibition that is enhanced when combined with the CCB effect, which indicate a therapeutic advantage of multi-action drugs for r-mTBI.

**Electronic supplementary material:**

The online version of this article (10.1186/s40478-020-01045-x) contains supplementary material, which is available to authorized users.

## Introduction

Globally, 61 million individuals are estimated to sustain Traumatic brain injury (TBI) each year [5]. In the United States alone, TBI affects over 2.5 million people annually, with mTBI representing 80% of all TBI cases, and to date accounts for more than 13 million cases of long-term disability [32]. Repeated exposure to mTBI (r-mTBI) has been to linked to higher risks of chronic neurodegenerative disorders including Alzheimers disease (AD) and Chronic Traumatic Encephalopathy (CTE) [4, 6, 12, 13, 31]. In fact, the pathologies of late r-mTBI share similar features with AD and CTE and include a marked neuroinflammatory response, the presence of neurofibrillary tangles (NFTs), and to a lesser extent, the presence of amyloid-$\beta$ (A$\beta$) pathology [9, 14, 30, 31]. Among them, neuroinflammation is a key element of both acute and chronic phases of r-mTBI and may represent a target for early therapeutic intervention.

In our previous pre-clinical work on AD, we have shown that the drug nilvadipine can reduce inflammation, decrease tau phosphorylation, and enhance A$\beta$ clearance across the blood brain barrier (BBB), while improving cognitive functions [2, 23, 24]. Nilvadipine is a dihydropyridine (DHP) that blocks L-type calcium channels and exhibits anti-hypertensive properties [3]. Nilvadipine is a racemic mixture of two enantiomers: (+)-nilvadipine and (−)-nilvadipine. (+)-nilvadipine is a L-type calcium channel blocker responsible for the anti-hypertensive properties of nilvadipine while (−)-nilvadipine is deprived of anti-hypertensive effect [23, 24]. Interestingly, we have shown that both enantiomers of nilvadipine are able to reduce neuroinflammation, tau hyperphosphorylation, and A$\beta$ production via a mechanism which appears to be independent of L-type calcium channel inhibition and mediated by SYK inhibition [23]. In a small clinical trial, nilvadipine delayed clinical progression of MCI to AD [8] while in an open label Phase I/IIa clinical study, nilvadipine showed safety and tolerability in the AD patients and no adverse effects on blood pressure [10]. More recently, in a Phase III randomized, placebo-controlled clinical trial for AD (NILVAD, n = 510), the very mild AD cases (≥ 25, N = 80) demonstrated a significant reduction in cognitive decline following treatment with nilvadipine compared to placebo [1, 11].

Our preclinical data, which demonstrated the ability of nilvadipine to suppress these pathologies common to both AD and TBI, encouraged us to test this drug in our models of r-mTBI. Earlier, we developed a r-mTBI model that successfully recapitulates aspects of human TBI pathology such as inflammation, axonal injury, and cognitive deficits [18–20]. We further tested a 21-day treatment with nilvadipine in the very old r-mTBI hTau mice to mimic brain traumas occurred in the elderly population [17]. We chose hTau mice because they express all 6 isoforms of human tau making it a convenient model for mimicking TBI-induced tauopathy, which is often observed in the elderly. Interestingly, nilvadipine ameliorated inflammation, tau phosphorylation and short-term memory deficits induced by r-mTBI. However, in the very old mice (24–26 months old), age-driven pathology precluded the differentiation of the injury effects from age effects. Therefore, in this study, we aimed to deliver a similar 21-day treatment with nilvadipine in young hTau mice and to assess pathological and cognitive response at 3 weeks post last mTBI. We also included treatment with the non-vasoactive (non-calcium-channel blocker) (−)–nilvadipine enantiomer (internal reference name ARC031) to investigate whether treatment effects on TBI outcomes required the ${\hbox {Ca}}^{2+}$-channel blocking effects of racemic nilvadipine.

## Methods and materials

### Animals

Male and female hTau mice 12–14 weeks old (weight 19–25 g) were sourced from Jackson Laboratories (Bar Harbor, ME). The animals were housed under standard laboratory conditions (14-h light/10-h dark cycle, 23 ± 1C, 50 ± 5% humidity) with free access to food and water. All procedures were carried out under Institutional Animal Care and Use Committee (IACUC) approval and in accordance with the National Institutes of Health Guide for the Care and Use of Laboratory Animals.

### Experimental groups and study design

**Fig. 1:**
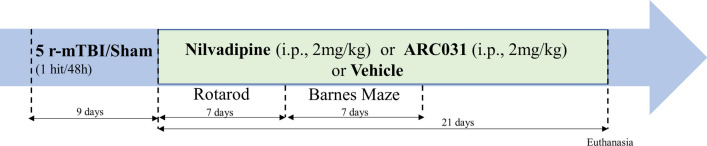
Study design

A total of 72 mice were randomly assigned to 1 of 6 groups (n = 12 per group): repetitive sham/vehicle, repetitive sham/nilvadipine, repetitive sham/ARC031, repetitive injury/vehicle, repetitive injury/nilvadipine and repetitive injury/ARC031. The r-mTBI (total of 5 hits with an inter-concussion interval of 48 h) was administrated to mice as previously described [18, 19, 21, 33, 34]. Sham injured animals (5 anesthesia, 48 h apart) underwent the same procedures and were exposed to anesthesia for the same length of time as the mTBI animals. Either nilvadipine, ARC031 or vehicle (PBS:PEG/1:1) were injected intraperitoneally (100 μL, i.p.) daily for 21 days, with the first injection administered immediately following the last injury. The behavior analysis began 24 h after the last mTBI/anesthesia for each group, as shown in Fig. 1. Euthanasia was performed 22 days after the last mTBI/sham procedure. Researchers were blind to animal group assignments during both neurobehavioral experiments and immunohistochemistry.

### Injury protocol

All animals were anesthetized with 1.5 L/min of oxygen and 3% isoflurane prior to r-mTBI or sham injury. The heads were shaved, and mice were placed on a heating pad to maintain body temperature at 37C to prevent hypothermia. The head of each animal was fixed in a stereotaxic frame, and the blunt impactor tip (3 mm diameter) was positioned midway to the sagittal suture. The injury was triggered at 5 m/s velocity and 1.0 mm depth, with a dwell time of 200 ms, using a myNeuroLab controller device (Impact ${\hbox {One}}^{\mathrm{TM}}$ Stereotaxic Impactor, Richmond, IL). All mice experienced short-term apnea ($<20$ s) and showed no skull fractures. All animals were allowed to recover from anesthesia on a heating pad and then returned to their cages with water and soft food access. Sham animals received anesthesia alone for the same duration of time as the r-mTBI mice, to control for the effects of repeated anesthesia. Mice were monitored daily for any abnormalities in behavior.

### Treatment

For convenience, racemic nilvadipine will be referred to as “nilvadipine”, while (−)-nilvadipine enantiomer as “ARC031” . All mice received either nilvadipine, ARC031 or vehicle via i.p. injections for 21 days, starting immediately after the last injury/sham (the first injection was administered while animals were still under anesthesia). The treated groups received 2 mg/kg nilvadipine (equivalent to a human oral dose of 8 mg conferring antihypertensive activity) or 2 mg/kg ARC031 dissolved in a 1:1 solution of PBS and PEG vehicle solution [23]. The injection volume (100 μL) was calculated based on the average animal’s weight (0.028 kg). Untreated animals underwent the same procedure but received vehicle solution only (PBS:PEG/1:1). All solutions were freshly prepared every day before the injections.

### Motor and cognitive function assessment

Motor function was assessed using the Rotarod apparatus, and the latency to fall from an accelerating rotating rod was measured. Baseline performance was recorded 1 day prior to the first injury/sham. Rotarod assessment started on the day after the last injury/sham procedure and was carried out on days 1, 3, 5 and 7 post-last r-mTBI/sham. An acclimation period involved 3 trials with a duration of 3 min each and a 3 min rest interval in the animal’s home cage between the trials (velocity = 5 rpm, no acceleration). The mice were placed back on the bar during the acclimation period if they fell. All experimental trials, including the baseline trials, lasted for 5 min and were conducted with acceleration from 5 to 50 rpm over the 5 min period. Each animal underwent 3 trials per day, with a 3 min rest interval between each trial. The fall time of each mouse was recorded in seconds. To ensure that fall time would correlate with motor coordination, rather than purely grip strength, if a mouse clung to the bar for more than 5 consecutive rotations on the accelerating rod without walking or making forward progress against the rotation of the bar, the time of the $5{\mathrm{th}}$ rotation was considered to be the fall time and was recorded as such.

Barnes Maze (BM) was initiated on day 8 post-last mTBI/sham and lasted for 7 consecutive days to assess cognitive function. For 6 days, animals were trained to find the target hole which had a black escape box underneath. The walls in the room were equipped with visual cues and the brightness of the room was consistent throughout testing (7 days). The BM table was 1.2 m in diameter and has 18 equally spaced holes around the perimeter. Every mouse had 4 acquisition trials per day, with a duration of 1.5 min each. The starting position for each trial during acquisition rotated, beginning at one of 4 cardinal directions of the maze and rotating, first clockwise (until reaching the initial position again) and then counterclockwise. If an animal did not find the target hole or did not go inside the box within the time limit, the mouse was guided to the target hole by hand. Regardless of their success, mice then spent 30 s in the box before returning to their cage. On the last day, 24 h following the final acquisition trial, a probe trial was conducted during which the animals were placed in the middle of the maze and had 60 s to find the target hole, from which the escape box had been removed. The cumulative distance from the target hole, total distance travelled, time to find the target hole, and velocity were calculated using Noldus Ethovision XT software and analyzed to assess spatial memory and learning. Cumulative distance was measured as the sum of the distance between the center point of the mouse and the center of the target hole for every video frame from each trial at 30 frames per second. This distance stopped accumulating when the trial ended, either when the total time had elapsed or when the mouse entered the target box. Data were presented as the raw values of the cumulative distance.

### Tissue collection and processing

Animals were euthanized on the day after the last injection (22 days post-last injury/sham). Mice were anesthetized with 3$\%$ isoflurane and perfused transcardially with phosphate-buffered saline (PBS), pH-7.4. After perfusion, the brains were post-fixed in a solution of 4% paraformaldehyde (PFA) at 4C for 48 h and paraffin-embedded for immunohistochemistry. Brain samples fixed in PFA were processed in paraffin using the Tissue-Tek VIP (Sakura, USA). Sagittal sections were cut at 8 μm using a Leica RM2235 microtome and mounted on positively charged glass slides. Prior to staining, sections were deparaffinized in xylene and rehydrated in ethanol solutions of decreasing concentrations.

### Immunohistochemistry

#### Non-fluorescent staining for GFAP

Following rehydration, slides were processed with hydrogen peroxide for 15 min and heated in citric acid buffer (pH-6) for antigen retrieval. Slides were then blocked with normal goat serum, washed with PBS, and incubated in a primary antibody for GFAP overnight at 4C (GFAP7857983, Aves Labs, Inc., 1:10,000). On the next day, slides were processed using the anti-chicken VectaSTAIN ABC Kit and developed with 3,3′-Diaminobenzidine (DAB) before mounting.

#### Non-fluorescent staining for Iba1

Microglia were stained using an anti-Iba1 antibody (ab107159, Abcam). After rehydration, slides were processed with hydrogen peroxide for 15 min followed by antigen retrieval using citric acid buffer (pH-6). Next, slides were blocked with rabbit serum for 1 h at room temperature and then incubated with the primary antibody (1:1000) overnight. On the next day, samples were processed using the anti-goat VectaSTAIN ABC Kit and developed with DAB.

### Fluorescent staining

#### GFAP/Iba1/CD68/P-SYK

Fluorescent staining was performed using the following antibodies: Iba1 (ab107159, Abcam), GFAP (7857983, Aves Labs), CD68 (ab125212, Abcam) and phosphorylated spleen tyrosine kinase P-SYK (Tyr525/526) (2710S, Cell Signaling). Following rehydration, antigen retrieval was performed by heating slides in citric acid buffer for 7 min in a microwave oven. Next, slides were washed with PBS and transferred to a Sudan Black solution for 15 min to prevent autofluorescence. Slides were then blocked for 1 h with 10% donkey serum solution in PBS, and primary antibodies for Iba1 (1:300), GFAP (1:1000), CD68 (1:500) and P-SYK (1:200) were applied overnight. On the next day, secondary antibodies AlexaFluor488 (A21202, Life Technologies), AlexaFluor647 (A21449, Life Technologies), AlexaFluor568 (ab175477, Abcam), were applied for P-SYK, GFAP, and Iba1/CD68, respectively. Slides were mounted with ProLong Gold Antifade 4′,6-diamidino-2-phenylindole (DAPI) Mount. Each marker was stained separately except for the double staining for Iba1/P-SYK that was performed to evaluate colocalization of the two. Fluorescent imaging was performed using a confocal microscope (LSM 800 Zeiss) at 20× and 63× magnification. Z-stacks were recorded for every image and orthogonal projections were obtained to enable a 3D representation of the picture.

### Immunohistochemical quantification

Imaging of *non-fluorescent* samples stained for GFAP/Iba1 was performed on an Olympus DP72 microscope at 10× magnification. Further analysis of the images included quantification of GFAP and Iba1 signal using ImageJ. Images were separated into individual color channels (hematoxylin counterstain and DAB chromogen) using the color deconvolution algorithm. Three nonoverlapping regions of interest (ROI) of 100 μm^2^ per image were then selected for the hippocampus, the body of the corpus callosum and the cortex. A coverage area ($\%$) per ROI was calculated and the mean value for each animal was used for further statistical analysis.

### MesoScale discovery (MSD) multi-spot assay

A tau profile was assessed in cortical mouse homogenates using MSD protocol using the Phospho (Thr231)/Total Tau kit (V-PLEX K15121D) following manufacturer’s instructions. First, the calibration solution Tau441 was prepared according to the kit protocol. Samples and controls were diluted twofold in Tau441. Then, the plate was blocked with Blocker A for 1 h followed by adding the samples and calibrators into the wells. After 1 h of incubation, the plate was incubated for 1 h with detection antibodies (SULFO-TAG), and the Read Buffer T was added to analyze the plate. The plate was washed with the Tris Wash Buffer after each step. The plate analysis was conducted on the MSD instrument (MESO Quick Plex SQ120).

### Statistical analysis

All experimental data were analyzed using JMP 12 and GraphPad Prism 6 software. The data were checked for normality using Skewness-Kurtosis and Goodness of Fit. If normal, parametric method one-way ANOVA was applied to calculate the significance in the tested groups (*p* values less than 0.05 were considered significant). If significant, post-hoc analysis was applied using Turkey’s multiple comparison test/Honest Significant Difference (HSD). The Turkey’s test compares all possible pairs of means between different treatment groups and was considered significant if $p< 0.05$. The Shapiro–Wilk test was used if data were not normally distributed. All data were transformed to logarithm or square root, when required, to reach normality before further analysis. Repeated-measure analysis of variance (MANOVA) was used to analyze continuous performance of mice in the Barnes Maze and Rotarod ($p<0.05$ is significant). Error bars represent the standard error of the mean.

## Results

### Motor assessment

**Fig. 2:**
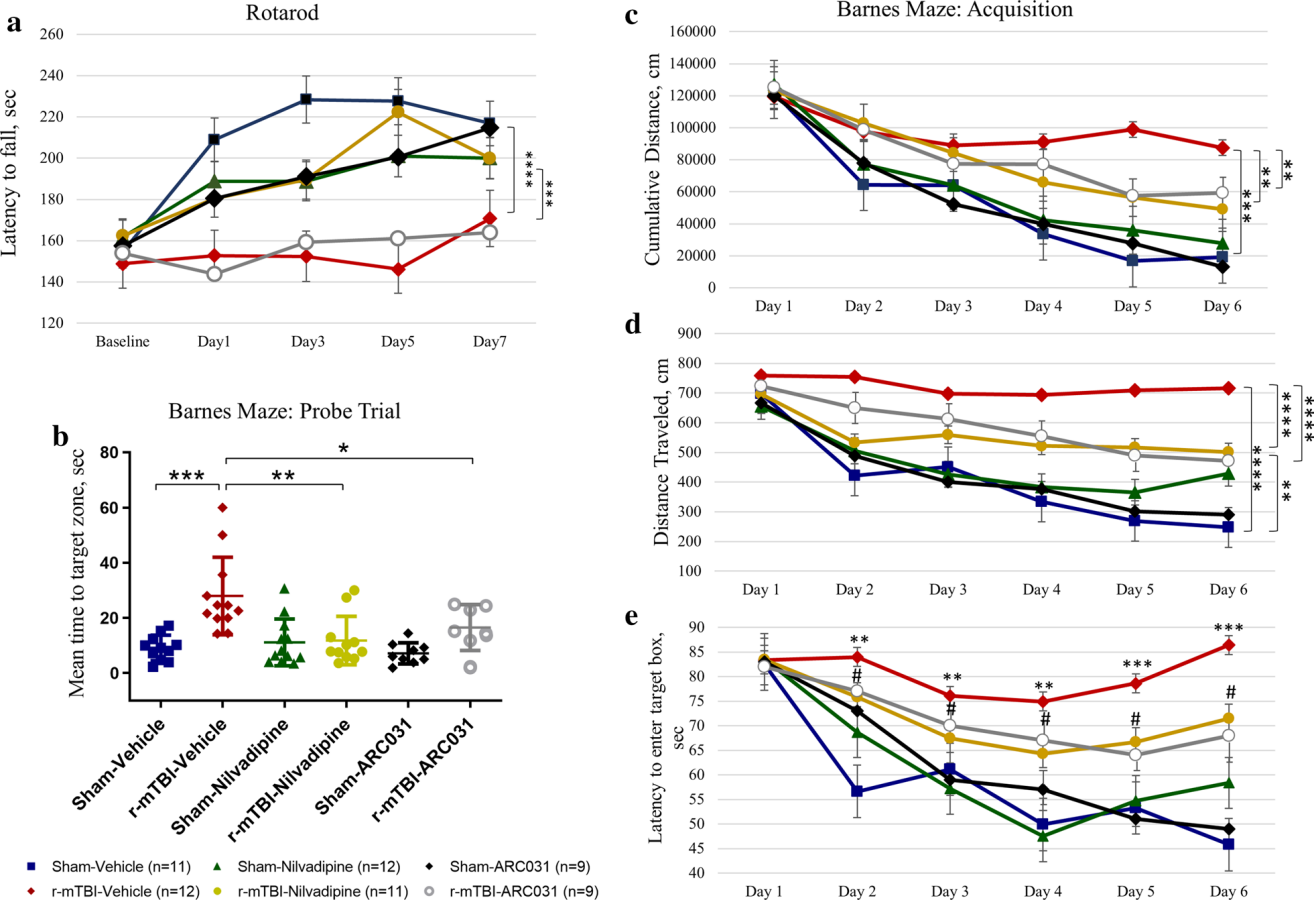
**a** Evaluation of the effect of nilvadipine or ARC031 on motor performance. Mice in the r-mTBI-vehicle group showed a significant decrease in their performance compared to the sham-vehicle ($p<0.001$). Treatment with nilvadipine ameliorated motor deficits in the r-mTBI mice (r-mTBI-nilvadipine vs. r-mTBI-vehicle, $p<0.001$). In r-mTBI mice treated with ARC031, no differences were observed in the latencies compared to the r-mTBI-vehicle ($p>0.05$). **b**–**e** Evaluation of spatial learning and memory. **b** Probe data show that r-mTBI-vehicle mice took more time to locate the target hole compared to the sham-vehicle controls ($p<0.05$). Both nilvadipine ($p<0.01$) and ARC031 ($p<0.05$) treatments in the r-mTBI mice ameliorated impaired memory functions compared to r-mTBI-vehicle ($p<0.05$). **c**–**e** Compared to sham-vehicle, r-mTBI-vehicle mice exhibit learning deficits during the 6 days of acquisition. All injured sham mice travelled greater distance (**c**, $p<0.001$; **d**, $p<0.001$) and failed to enter the box (**e**, days 2–4, $p<0.01$, days 5–6 $p<0.001$) compared to sham-vehicle mice. R-mTBI-nilvadipine/ARC031 mice showed an improvement in all 3 parameters compared to the r-mTBI-vehicle (**c** cumulative distance $p<0.01$/$p<0.01$, **d** distance traveled $p<0.0001$/$p<0.0001$, **e** latency to target box $p<0.05$/$p<0.05$). Distribution (M-male, F-female): sham-vehicle n = 11 (5M, 6F), r-mTBI-vehicle n = 12 (6M, 6F), sham-nilvadipine n = 12 (6M, 6F), r-mTBI-nilvadipine n = 11 (6M, 5F), sham-ACR031 n = 9 (5M, 4F), r-mTBI-ARC031 n = 9 (5M, 4F). Data are presented as mean $\pm$ standard error of the mean; significance was calculated using one-way ANOVA and MANOVA

Motor functions were assessed using Rotarod on days 1, 3, 5 and 7 post-last TBI. Overall, all mice except the r-mTBI-vehicle group exhibited at least 40% increase in latency to fall over a 7-day period (Fig. 2a). R-mTBI-vehicle mice did not demonstrate improvement in their performance when compared to the baseline. Nilvadipine treatment in the injured mice ameliorated their motor deficits to levels not significantly different from sham mice and did not affect the behavior in healthy sham mice (r-mTBI-vehicle vs. sham-vehicle $p<0.0001$, r-mTBI-nilvadipine vs. r-mTBI-vehicle $p< 0.001$, sham-nilvadipine vs. sham-vehicle $p> 0.05$, r-mTBI-nilvadipine vs. sham-vehicle $p> 0.05$, MANOVA). Treatment with ARC031 did not show an amelioration of the locomotor deficits in the r-mTBI mice ($p> 0.05$, MANOVA).

### Memory assessment

Spatial learning and memory were assessed using Barnes Maze during days 8–14 post last TBI and the probe trial was recorded on day 15. Sham-vehicle mice demonstrated an 83% decrease of the cumulative distance and a 71% decrease in the distance traveled from day 1 to 6 (Fig. 2c, d). R-mTBI vehicle mice exhibited a 25% reduction of the cumulative distance during days 1–3 which reached a plateau on days 4–6 (r-mTBI-vehicle: day1 vs. day6 $p<0.05$, one-way ANOVA). R-mTBI-vehicle mice had significantly higher cumulative distance (vs. sham-vehicle $p<0.001$, MANOVA) and distance traveled (vs. sham-vehicle $p<0.0001$, MANOVA) than controls. Travel distance in r-mTBI-vehicle mice stayed consistent with day 1 during an entire acquisition period (r-mTBI-vehicle: day1 vs. day6 $p>0.05$, one-way ANOVA). On days 2–6, r-mTBI-vehicle mice traveled for a longer time than sham-vehicle (r-mTBI-vehicle vs. sham-vehicle: for day2 and day3 $p<0.01$; for day4, day5, and day6 $p<0.001$, one-way ANOVA). Treatment with nilvadipine in r-mTBI mice lead to a 44% decrease in cumulative distance (r-mTBI-nilvadipine vs. r-mTBI-vehicle $p< 0.01$, r-mTBI-nilvadipine vs. sham-vehicle $p>0.05$, MANOVA) and a 28.6% decrease in the distance traveled (r-mTBI-nilvadipine vs. r-mTBI-vehicle $p< 0.0001$, r-mTBI-nilvadipine vs. sham-vehicle $p<0.01$, MANOVA). Similarly, a decrease in the latency to enter the target box from day 1 to day 6 was observed for sham-vehicle (44%) and sham-nilvadipine (25%) groups (Fig. 2e). After r-mTBI, mice took 50% longer to locate the box compared to the sham-vehicle, with only transient improvements on days 3–5. By day 6, a 35% increase in time to locate the box was recorded for the r-mTBI-nilvadipine mice compared to sham-vehicle. This time was still notably decreased by 20% when r-mTBI-nilvadipine are compared to r-mTBI-vehicle. Similarly, treatment with ARC031 in r-mTBI mice decreased cumulative distance (r-mTBI-ARC031 vs. r-mTBI-vehicle, $p<0.01$, MANOVA), distance traveled (r-mTBI-ARC031 vs. r-mTBI-vehicle, $p<0.0001$, MANOVA) and latency (r-mTBI-ARC031 vs. r-mTBI-vehicle: for day2 $p<0.01$, for day3 and day4 $p<0.05$, for day5 and day6 $p<0.01$, one-way ANOVA) compared to vehicle-treated r-mTBI groups (Fig. 2c–e). During the probe trial, r-mTBI-vehicle mice spent a significantly longer time locating the target hole indicating deficits in spatial memory (Fig. 2b; r-mTBI-vehicle vs. sham-vehicle $p<0.05$, one-way ANOVA). Nilvadipine recovered these deficits back to the control levels with no effects on healthy sham mice (r-mTBI-nilvadipine vs. r-mTBI-vehicle $p< 0.01$, r-mTBI-nilvadipine vs. sham-vehicle $p>0.05$, one-way ANOVA). ARC031 also decreased r-mTBI-induced memory impairments during the probe trial (Fig. 2b; r-mTBI-ARC031 vs. r-mTBI-vehicle $p< 0.05$, r-mTBI-ARC031 vs. sham-vehicle $p>0.05$, one-way ANOVA).

### Immunohistochemistry

**Fig. 3:**
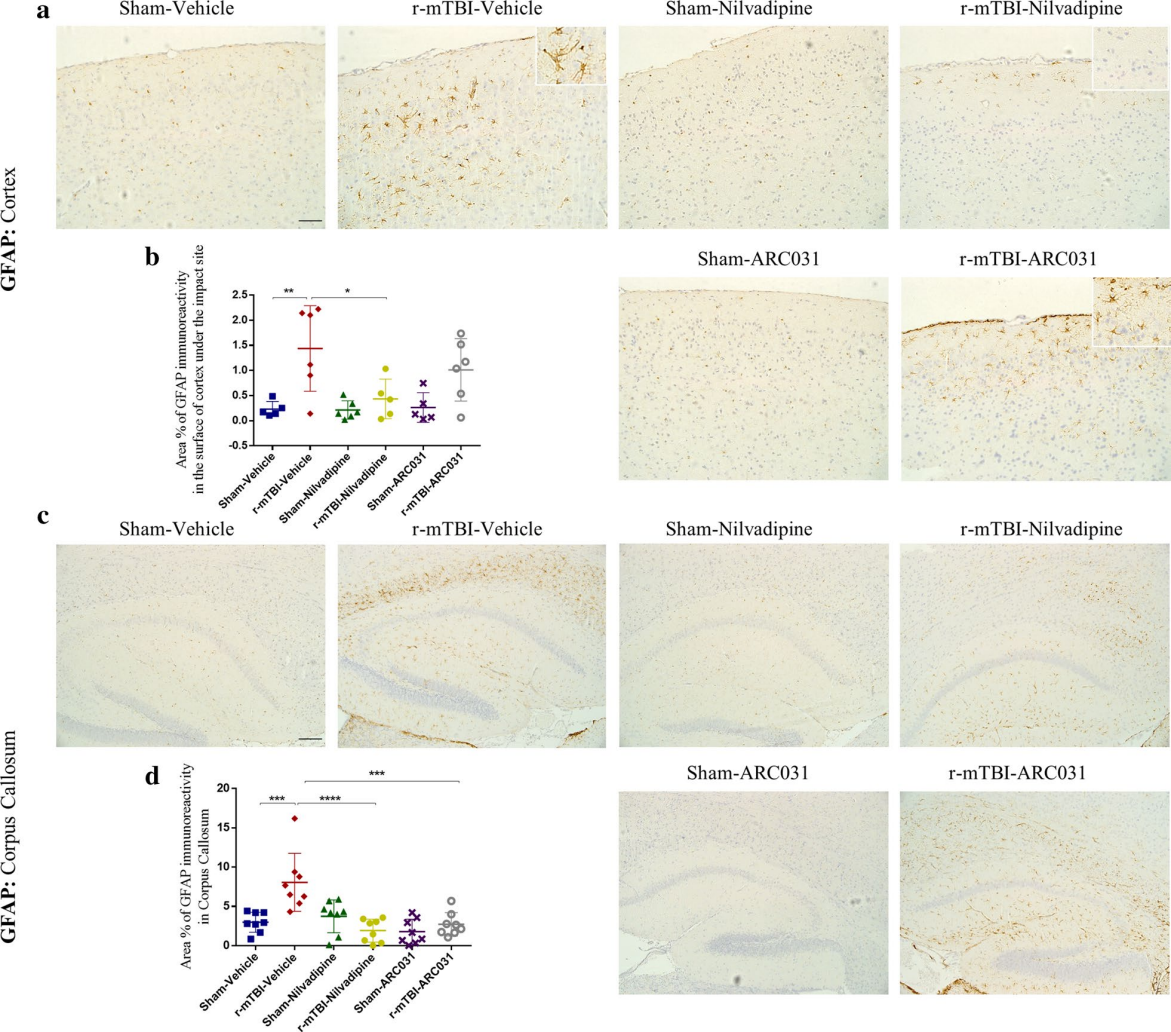
Evaluation of the effects of nilvadipine or ARC031 on astrocytes activation. **a**–**b** An increase in the area of GFAP staining was observed in the r-mTBI-vehicle mice compared to sham-vehicle ($p<0.001$). This increase was suppressed in the r-mTBI-nilvadipine mice ($p<0.05$). **c**–**d** In the corpus callosum, r-mTBI-vehicle mice also exhibited elevated levels of GFAP compared to the sham-vehicle mice ($p<0.001$). Treatment with nilvadipine and ARC031 in the r-mTBI mice reduced astrogliosis ($p<0.0001$, $p<0.001$, respectively) compared to respective TBI-vehicle. Distribution of n “cortex/hippocampus” (M-male, F-female): sham-vehicle n = 5/8 (3M, 2F/4M, 4F), r-mTBI-vehicle n = 6/8 (3M, 3F/4M, 4F), sham-nilvadipine n = 6/8 (3M, 3F/4M, 4F), r-mTBI-nilvadipine n = 5/8 (2M, 3F/4M, 4F), sham-ACR031 n = 5/8 (2M, 3F/4M, 4F), r-mTBI-ARC031 n = 6/8 (3M, 3F/4M, 4F). Data are presented as mean $\pm$ standard error of the mean; significance was calculated using one-way ANOVA. Scale bars equal 200 μm

**Fig. 4:**
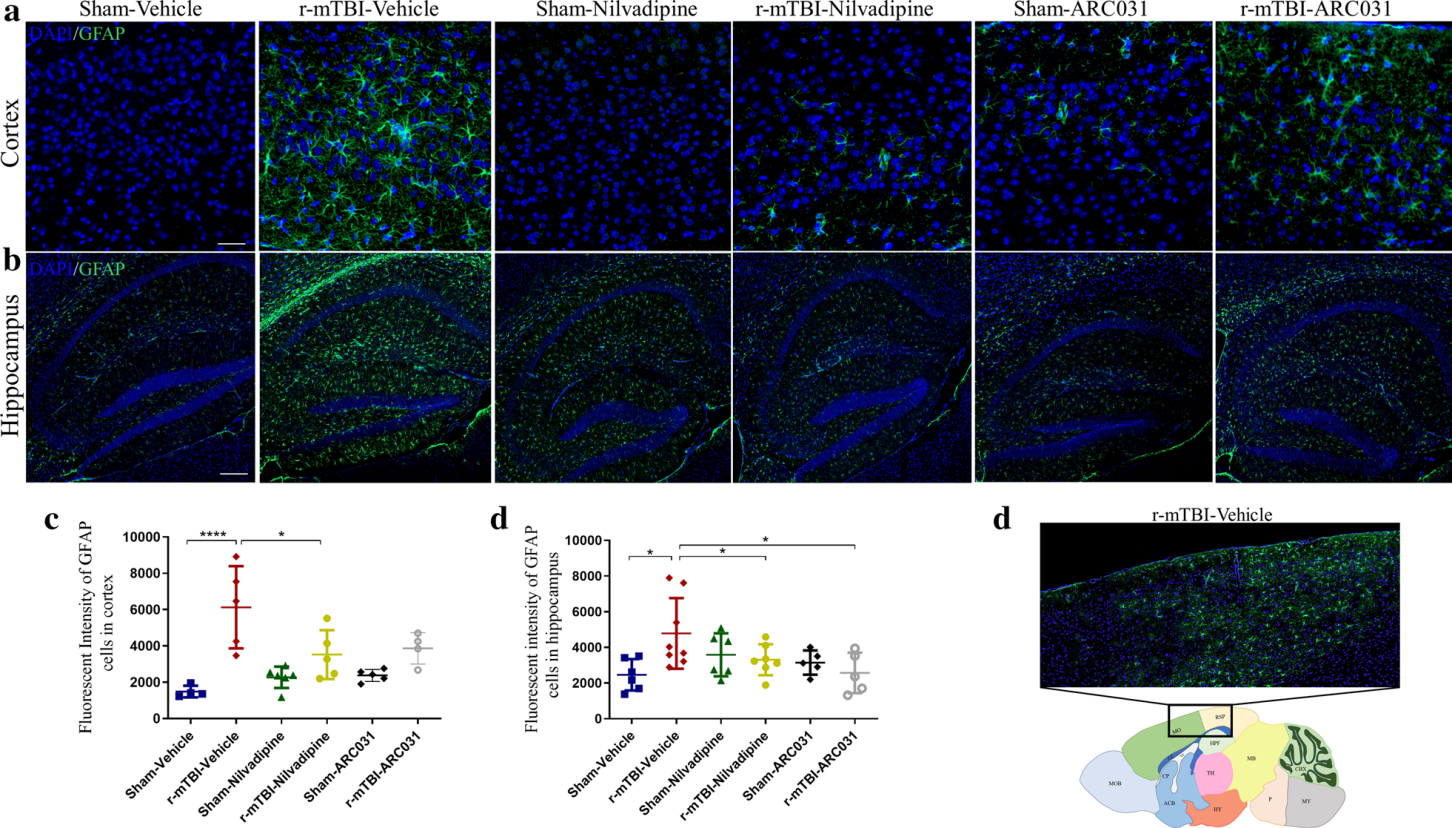
Immunofluorescent images of GFAP signal in cortex (**a**) and hippocampus (**b**). In the r-mTBI-vehicle mice, strong GFAP signal was found in cortex under the injury site spreading into the deeper layers including retrosplenial and somatomotor cortical regions (**a**, **e**). In r-mTBI-nilvadipine mice, no GFAP signal was found in similar cortical (**a**, **c**) or hippocampal regions (**b**, **d**). In r-mTBI-ARC031 mice, GFAP signal was significantly reduced in hippocampus (**b**, **d**), but not in cortex (**a**, **c**), compared to the r-mTBI-vehicle group. Cortical GFAP in r-mTBI-ARC031 was not significantly different from sham-vehicle (**d**). Distribution of n in “cortex/hippocampus” (M-male, F-female): sham-vehicle n = 4/6 (2M, 2F/3M, 3F), r-mTBI-vehicle n = 5/8 (3M, 2F/5M, 3F), sham-nilvadipine n = 6/6 (3M, 3F/4M, 2F), r-mTBI-nilvadipine n = 5/7 (2M, 3F/3M, 4F), sham-ACR031 n = 5/5 (2M, 3F/3M, 2F), r-mTBI-ARC031 n = 4/5 (2M, 2F/4M, 1F). Data are presented as mean $\pm$ standard error of the mean; significance was calculated using one-way ANOVA. Scale bars equal 20 μm for cortex and 200 μm for hippocampus

GFAP staining revealed no gliosis in the healthy tissue of sham-vehicle mice (Fig. 3a–d). An increased astroglial reactivity was prominent in the r-mTBI-vehicle mice in the corpus callosum ($p<0.05$, one-way ANOVA) and in the retrosplenial (RSP) and somatomotor (MO) areas of the cortex surrounding the injury site ($p<0.001$, one-way ANOVA) (Figs. 3a–d, 4a, c, d). This r-mTBI induced increase was reduced by nilvadipine treatment in all three analyzed brain areas (corpus callosum $p<0.01$, hippocampus $p<0.05$, cortex $p<0.05$, one-way ANOVA). In the r-mTBI-ARC031 mice, a significant decrease of astrogliosis was also observed in the corpus callosum ($p<0.001$, one-way ANOVA) but not in the cortex ($p>0.05$, one-way ANOVA). Immunofluorescent analysis confirmed a significant decrease of astrogliosis in the superficial layers of the cortex in the r-mTBI-nilvadipine versus r-mTBI-vehicle mice but not in r-mTBI-ARC031 mice (Fig. 4a, c). Moreover, a decreased fluorescent intensity for astroglial marker GFAP was shown in the hippocampus in both r-mTBI-nilvadipine and r-mTBI-ARC031 cohorts compared to r-mTBI-vehicle controls (Fig. 4b, d).

**Fig. 5:**
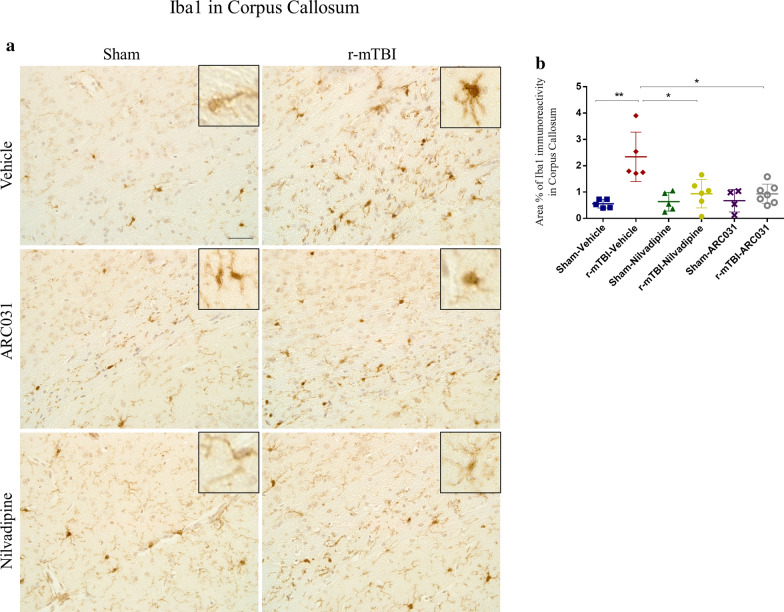
**a**–**b** Effects of nilvadipine on microglia in the r-mTBI mice. After r-mTBI, Iba1 signal was increased in the CC compared to sham-vehicle ($p<0.01$). A significant decrease of Iba1 was shown in the r-mTBI mice after treatment with nilvadipine ($p<0.05$) and ARC031 ($p<0.01$). Distribution (M-male, F-female): sham-vehicle n = 5 (2M, 3F), r-mTBI-vehicle n = 5 (1M, 4F), sham-nilvadipine n = 5 (3M, 2F), r-mTBI-nilvadipine n = 6 (4M, 2F), sham-ACR031 n = 4 (2M, 2F), r-mTBI-ARC031 n = 7 (5M, 2F). Data were analyzed using one-way ANOVA. Scale bars equal 20 μm

IHC staining for Iba1 revealed a 50% increase in microgliosis in the r-mTBI-vehicle mice compared to sham-vehicle mice in the corpus callosum ($p<0.01$, one-way ANOVA) but not in the cortex ($p>0.05$, one-way ANOVA) (Fig. 5a, b). In the r-mTBI-nilvadipine mice, Iba1 showed a 50% decrease (to close to sham levels) compared to the r-mTBI-vehicle mice ($p<0.05$, one-way ANOVA). Similarly, treatment with ARC031 reduced Iba1 immunostaining in the corpus callosum of r-mTBI-ARC031 mice compared to r-mTBI-vehicle ($p<0.01$, one-way ANOVA).

**Fig. 6:**
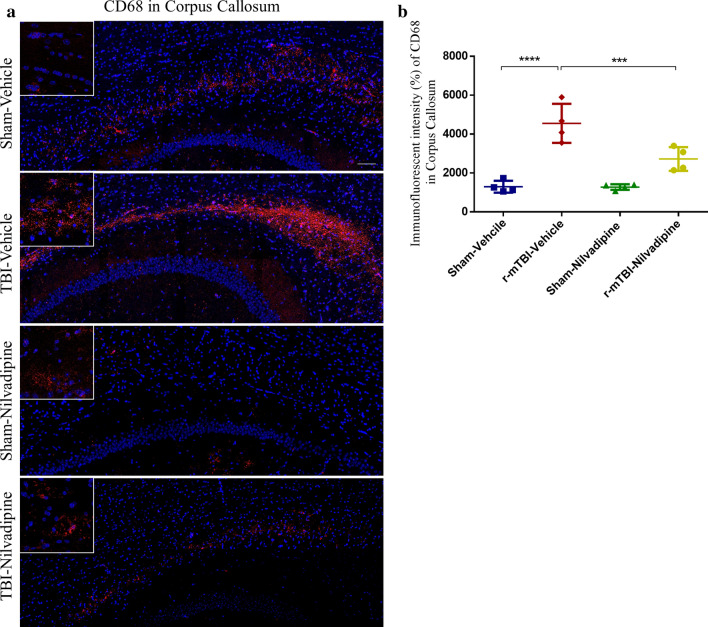
**a** Immunofluorescent imaging of CD68 in corpus callosum. **b** Quantification of the fluorescent intensity of CD68 signal demonstrated an increased distribution of CD68 macrophages in the TBI-vehicle mice compared to controls ($p<0.0001$) and a decreased signal after treatment with nilvadipine ($p<0.001$). Distribution (M-male, F-female): sham-vehicle n = 4 (2M, 2F), r-mTBI-vehicle n = 4 (1M, 3F), sham-nilvadipine n = 4 (1M, 3F), r-mTBI-nilvadipine n = 4 (2M, 2F). Data were analyzed using one-way ANOVA. Scale bars equal 100 μm

Immunofluorescence for CD68, a lysosomal protein expressed in high levels by macrophages and activated microglia and in low levels by resting microglia, showed an increased signal in the corpus callosum of r-mTBI mice compared to the sham-vehicle (Fig. 6, $p<0.0001$). Treatment with nilvadipine decreased CD68 in the r-mTBI mice compared to the vehicle treated TBI mice ($p<0.001$). Nilvadipine did not affect CD68 signal in the sham mice.

**Fig. 7:**
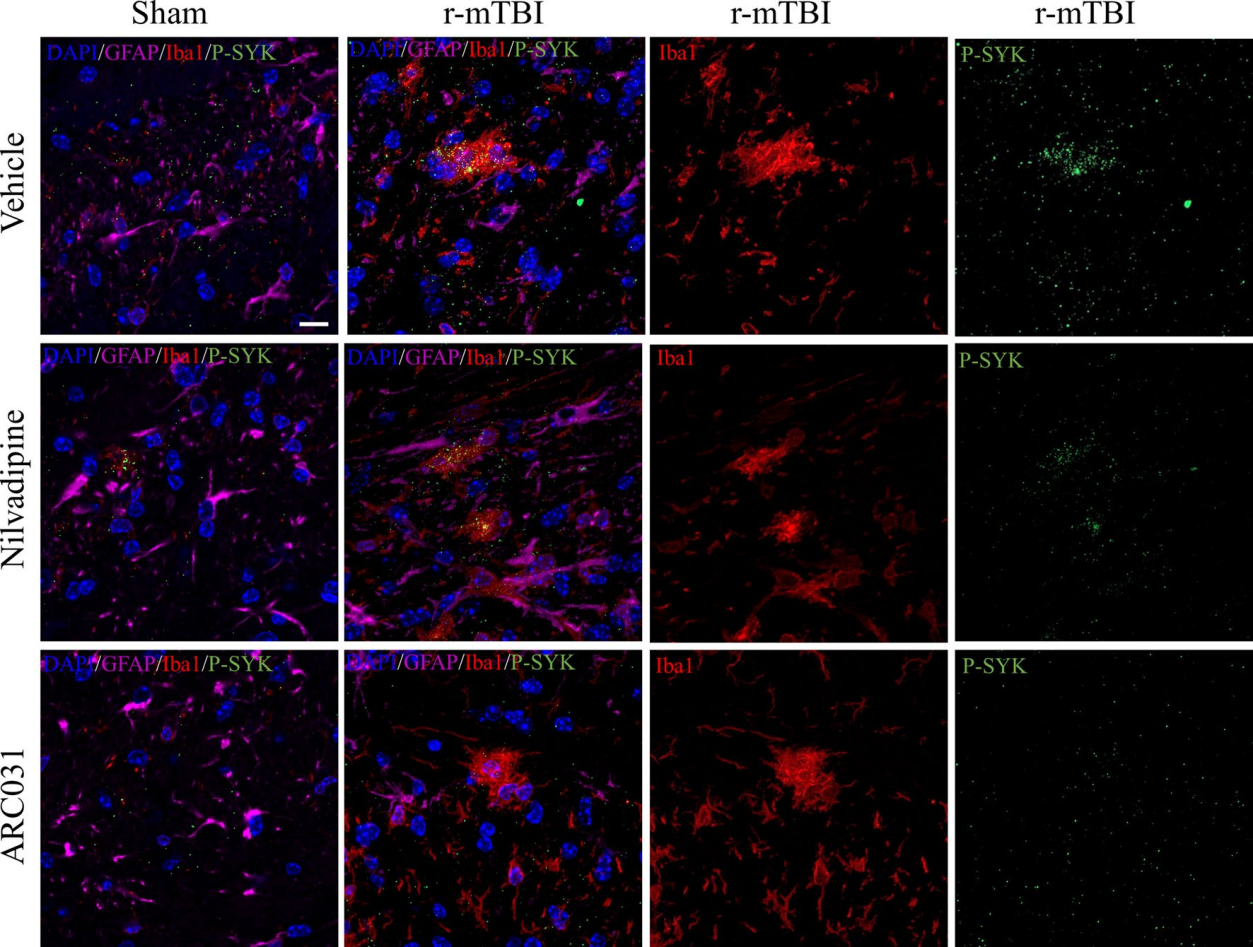
Representative images of GFAP, P-SYK and Iba1 in corpus callosum. No sham animals showed reactive astrocytes, microgliosis or signs of P-SYK. In the r-mTBI-vehicle mice, microglia appear to acquire amoeboid morphology and colocalize with P-SYK accumulations. Treatment with nilvadipine or ARC031 also eliminated detection of P-SYK in the corpus callosum in the areas near Iba1-reactive microglia. Distribution (M-male, F-female): sham-vehicle n = 10 (5M, 5F), r-mTBI-vehicle n = 12 (6M, 6F), sham-nilvadipine n = 11 (5M, 6F), r-mTBI-nilvadipine n = 10 (5M, 5F), sham-ACR031 n = 8 (4M, 4F), r-mTBI-ARC031 n = 7 (3M, 4F). Scale bars equal 10 μm

SYK response was measured by targeting the SYK phosphorylation site Tyr525/526, which is the main site involved in receptor-mediated SYK activation [27]. Fluorescent signal for P-SYK (Tyr 525/526) was detected in the r-mTBI-vehicle mice but absent in the young sham-vehicle mice (Fig. 7). Patchy P-SYK distribution was found in the corpus callosum of r-mTBI-vehicle mice but it was not easily detected and could not be quantified. The P-SYK images of the r-mTBI-vehicle group presented on Fig. 7 are representative of n = 12 analyzed samples. All detected areas of P-SYK accumulation were colocalized with Iba1-immunoreactive microglia. In the r-mTBI-nilvadipine and r-mTBI-ARC031 mice, there was no evidence for P-SYK in the corpus callosum as shown on the representative images from the total n = 10 and n = 7 analyzed images, respectively.

Tau levels were measured by immunoassay using MSD Phospho(Thr231)/Total Tau kit (Additional file 1). No r-mTBI-induced increase of total or p-tau was observed in cortical tissue compared to the sham-vehicle group. Similarly, treatment with nilvadipine in either sham or r-mTBI mice did not alter levels of p-tau compared to the respective controls. A significant decrease in total tau was observed in sham-nilvadipine (vs. sham-vehicle, $p<0.01$ and r-mTBI-vehicle, $p<0.001$) and in r-mTBI-nilvadipine (vs. r-mTBI-vehicle, $p<0.01$) mice.

## Discussion

Our model of r-mTBI demonstrates clear glial activation with motor and memory impairments in the first 21 days following the last injury. Areas with notable glial response included the corpus callosum (CD68, Iba1, GFAP) and cortical regions MO and RSP (GFAP). Interestingly, the retrosplenial (RSP) region, which is involved in spatial cognition, appears to show evidence of neuroinflammation after TBI in our model, together with the corpus callosum, and may therefore contribute to the observed cognitive impairment in our animals [15]. Treatment with nilvadipine attenuated both motor and memory deficits induced by r-mTBI and reduced astrogliosis (cortex, corpus callosum) and microgliosis (corpus callosum) to the levels observed in sham mice.

We have previously demonstrated that nilvadipine exhibits therapeutic properties by inhibiting SYK phosphorylation, which is linked to neuroinflammation, tau phosphorylation and cognitive deficits [23]. In AD mouse models, we have shown that SYK is activated in dystrophic neurites around amyloid deposits as well as in a subset of activated microglia, while SYK is upregulated in neurons displaying pathological tau species [27]. In our previous study, we showed that r-mTBI in aged mice caused patchy accumulation of P-SYK in the corpus callosum, usually colocalizing with amoeboid microglia, suggesting that SYK activation could play a crucial role in acute and sub-acute inflammation after mTBI [17]. Nilvadipine was able to reduce P-SYK in both mouse models of AD and aged r-mTBI [17, 23]. In the current study, P-SYK colocalized with microglia was also detected in the corpus callosum similar to the distribution pattern we previously observed in aged r-mTBI mice [17]. Nilvadipine decreased P-SYK signal in the r-mTBI mice in selected areas of corpus callosum and did not affect its signal in the sham mice. The results presented are based on the qualitative analysis, and the role of SYK in r-mTBI will be evaluated in future studies.

Previously, we have reported that nilvadipine successfully reduced phosphorylated tau in the aged r-mTBI mice [17], but it failed to do so in our current study. One possible reason may be the lack of r-mTBI-induced increase of p-tau due to the young age of mice (3 months old) which we have previously noted, although such increases are apparent in older hTau mice where tau pathology already exists [22]. Such age-dependent differences in p-tau in hTau mice may affect therapeutic response to nilvadipine. Nevertheless, nilvadipine did attenuate total tau levels in both sham and r-mTBI mice suggesting its ability to suppress total tau regardless of injury. Consistent with this, we have previously reported that SYK inhibition at 30 weeks of age (when tau pathology is prevalent) decreased total tau in P301S mice [28]. Schweig et al. demonstrated that inhibition of SYK did not affect *expression* of total tau but rather increased its degradation via an mTOR-dependent autophagy pathway [28].

The SYK inhibitory properties of nilvadipine have been previously demonstrated in mouse models of AD and tauopathies [23], but more work is required to confirm that this is the mechanism through which it elicits the favorable responses described in this study. Nilvadipine is also an L-type ${\hbox {Ca}}^{2+}$ channel blocker [26] and similar compounds have been proposed as having therapeutic benefit for TBI and have been investigated in several clinical trials (albeit these were more severe TBI cases) [35]. Prevention of calcium influx through secondary mechanisms was also shown to be effective in acute TBI pathology. For example, glutamate release following primary injury leads to rapid activation of NMDA receptors and calcium overload whereas ongoing secondary injury produces downregulation of NMDA receptor expression [7, 16]. In multiple TBI models, NMDA antagonists were able to prevent calcium overload when administered within 24–72 h post TBI [29] while treatment with NMDA agonists at acute timepoints (8 and 16 h post TBI) was not effective. Later administration of these agonists, however, improved synaptic plasticity and neurological functions (for review see [16]). However, a review of 8 clinical trials that tested CCBs in patients with severe TBI, traumatic subarachnoid hemorrhage (tSAH) and diffuse axonal injury (DAI) identified no significant beneficial effects of CCBs [35].

We have previously shown efficacy of the non-blood-pressure-lowering enantiomer of nilvadipine ((−)-nilvadipine/ARC031) in the controlled cortical impact mouse model of TBI (Ferguson, pers. comm). Here, we introduced the (−) enantiomer ARC031 into mice to test for the role of non-calcium channel blocking effects of nilvadipine. Interestingly, ARC031 showed a slightly different effect in the r-mTBI mice compared to racemic nilvadipine. First, it did not attenuate motor deficits, but it did improve memory impairment in the r-mTBI mice. Second, it reduced astrogliosis in the corpus callosum but, unlike nilvadipine, had no effect on reactive astrocytes in the cortex. It did, however, decrease microgliosis in the corpus callosum of r-mTBI mice. Third, it decreased SYK phosphorylation in the corpus callosum in a similar manner to nilvadipine. The lack of effect of (−) enantiomer ARC031 (compared to racemic nilvadipine) on motor outcomes may be linked to the effects of nilvadipine on cerebral blood flow or calcium-associated pathways; possibly the effects on cortical astrogliosis may also be related to vascular involvement of nilvadipine that is absent in ARC031. In a previous study in the TgAPPsw mouse model of Alzheimer’s disease, we treated 13-month old mice, which mimic AD-like pathology including reduced cerebral blood flow (CBF), with nilvadipine (1 mg/kg) daily for 15 days, and they demonstrated an increase in CBF compared to the vehicle-treated mice [25]. Thus, the positive effects of nilvadipine treatment after TBI could, at least in part, result from its cerebrovascular influence. Nilvadipine remains a plausible candidate compared to most other DHPs due to its ability to cross BBB and to inhibit SYK activation. Further studies will look into the use of other SYK inhibitors combined with anti-hypertensive drugs in our 5r-mTBI model to validate our hypotheses.

## Conclusion

Current work presents novel data that suggest that SYK could represent an effective therapeutic target in our preclinical model of r-mTBI, wherein SYK inhibition decreases TBI-dependent gliosis and improves cognitive and motor functions. Compared to the non-vasoactive (−)-nilvadipine, racemic nilvadipine appears slightly superior at mitigating behavioral and pathological impairments following TBI, suggesting that L-type calcium channel inhibition in addition to SYK inhibition contributed to the beneficial effects observed. These findings are important for the TBI field as they elaborate on potential therapeutic mechanisms and contribute to drug discovery research for brain trauma. Additional studies are ongoing to validate the contribution of SYK-dependent mechanisms in r-mTBI.

## Supplementary information

**Additional file 1.** Biochemical analysis of cortical p-tau (pThr-231) (A), total tau (DA9) (B) and the ratio of p-tau/total tau (C). No significant differences were shown between the cohorts for p-tau (A). Total tau was decreased in both sham-nilvadipine (p<0.001) and r-mTBI-nilvadipine (p<0.01) groups compared to r-mTBI-vehicle mice (B). A decrease in total tau was also recorded in sham-nilvadipine vs sham-vehicle mice (p<0.01) (B). The ratio of p-tau/total tau was increased in sham-nilvadipine mice compared to both sham-vehicle and r-mTBI-vehicle mice. Each cohort had n=8 (4M,4F). Data are presented as mean ± standard error of the mean; significance was calculated using one-way ANOVA.

## Data Availability

The datasets during and/or analyzed during the current study available from the corresponding author on reasonable request.
