# Supplementary figures and images for: Nilvadipine suppresses inflammation via inhibition of P-SYK and restores spatial memory deficits in a mouse model of repetitive mild TBI
Source: Acta Neuropathol Commun. 2020 Oct 19;8:166. doi: 10.1186/s40478-020-01045-x (PMC7574534; doi:10.1186/s40478-020-01045-x)

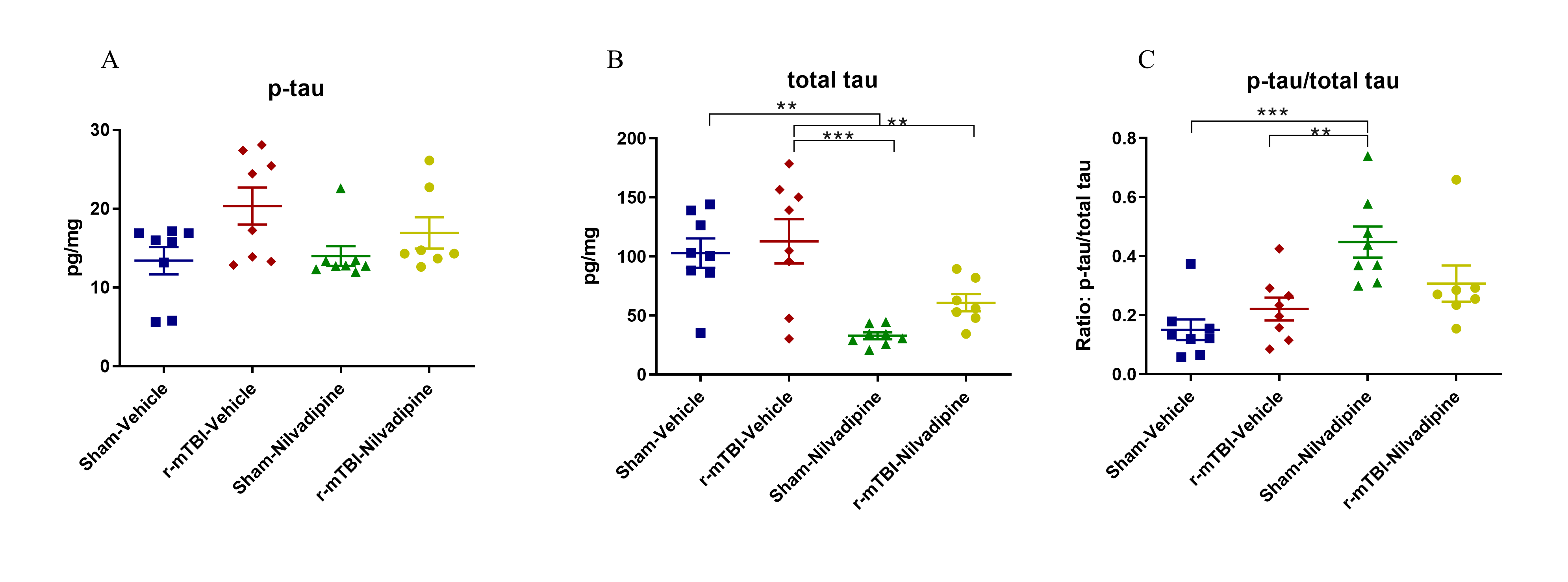

Supplement: Supplementary file 1 — Additional file 1. Biochemical analysis of cortical p-tau (pThr-231) (A), total tau (DA9) (B) and the ratio of p-tau/total tau (C). No significant differences were shown between the cohorts for p-tau (A). Total tau was decreased in both sham-nilvadipine (p<0.001) and r-mTBI-nilvadipine (p<0.01) groups compared to r-mTBI-vehicle mice (B). A decrease in total tau was also recorded in sham-nilvadipine vs sham-vehicle mice (p<0.01) (B). The ratio of p-tau/total tau was increased in sham-nilvadipine mice compared to both sham-vehicle and r-mTBI-vehicle mice. Each cohort had n=8 (4M,4F). Data are presented as mean ± standard error of the mean; significance was calculated using one-way ANOVA. [file 40478_2020_1045_MOESM1_ESM.tif]
